# Clinical considerations for medication-related osteonecrosis of the jaw: a comprehensive literature review

**DOI:** 10.1186/s40729-021-00323-0

**Published:** 2021-05-14

**Authors:** Mampei Kawahara, Shinichiro Kuroshima, Takashi Sawase

**Affiliations:** 1 Oral & Maxillofacial Implant Center, Nagasaki University Hospital, 852-8588, Nagasaki, Japan; 2 Department of Applied Prosthodontics, Graduate School of Biomedical Sciences, Nagasaki University, Nagasaki, 852-8588 Japan

**Keywords:** Medication-related osteonecrosis of the jaw, Antiresorptive agents, Bisphosphonates, Denosumab, Angiogenesis inhibitors

## Abstract

**Background:**

Medication-related osteonecrosis of the jaw (MRONJ), which was first reported as bisphosphonate-related osteonecrosis of the jaw (BRONJ) in bisphosphonate users, is a rare but severe soft and hard tissue disease induced by several types of medications. There has been a deluge of information about MRONJ, such as epidemiology, risk factors, clinical recommendations for dental treatment to prevent it, and treatment strategies in medication-prescribed users. The aim of this study was to comprehensively review recent articles and provide the current scientific information about MRONJ, especially clinical considerations or recommendations for dental treatment to prevent its occurrence.

**Materials and methods:**

The current literature review was mainly based on 14 systematic reviews with or without meta-analysis, 4 position papers, 1 consensus statement, 1 clinical guideline, and 2 clinical reviews regarding MRONJ after a PubMed database and manual searches according to inclusion and exclusion criteria. Moreover, 53 articles were selected by manual search in regard to all references from selected articles and other articles identified on the PubMed search, irrespective of publication date, and inclusion and exclusion criteria.

**Results:**

The incidence and prevalence of MRONJ are relatively low, although they are clearly higher in cancer patients receiving high-dose antiresorptive agents or angiogenesis inhibitors rather than osteoporosis patients receiving oral bisphosphonates or denosumab. There are many types of local, systemic, and other risk factors for the development of MRONJ. Clinical recommendations are provided for each clinical situation of patients to prevent MRONJ. There are also treatment strategies for MRONJ in each stage.

**Conclusions:**

General dentists should perform appropriate dental treatment to prevent MRONJ in the patients prior to or when receiving medications that could induce MRONJ. Moreover, there are treatment strategies for MRONJ in each stage that oral surgeons could follow. Adequate and updated clinical information regarding MRONJ based on scientific data is required whenever possible.

## Background

Medication-related osteonecrosis of the jaw (MRONJ) [1, 2], which includes bisphosphonate-related osteonecrosis of the jaw (BRONJ) [3], denosumab-related osteonecrosis of the jaw (DRONJ) [4, 5], and osteonecrosis of the jaw induced by angiogenesis inhibitors [6], is a rare but intractable disease. MRONJ has been shown to be one of the serious adverse side effects of antiresorptive agents such as bisphosphonates and anti-receptor activator of NF-kappa B ligand (denosumab) or angiogenesis inhibitors. Antiresorptive agents have been widely used to treat osteoporosis, hypercalcemia caused by malignant tumors, and skeletal-related events (SREs) such as bone pain and pathological fractures of vertebrae induced by multiple myeloma and solid tumors. Angiogenesis inhibitors such as the anti-vascular endothelial growth factor (VEGF) antibody agent bevacizumab have also been used for the treatment of several cancers. Recently, other medications such as neutralizing antibodies to tumor necrosis factor (TNF)-α, CD20, and sclerostin, and molecular targeted drugs, have been reported to possibly induce MRONJ [7], which indicates that the number of MRONJ patients has increased with the increased number of systemic diseases requiring antiresorptive agents and/or other medications each year.

MRONJ worsens oral health-related quality of life [8], prevents ideal dental treatment, and decreases activities of daily living, due to chewing disability and defects of jaw bones caused by surgical treatment and/or pathological progression of MRONJ. Therefore, clarifying the exact mechanisms of and establishing treatment strategies for MRONJ are imperative. However, scientific data about the pathophisiology of and definitive treatment strategies for MRONJ are limited. Indeed, sufficient scientific evidence for the current treatment methods has not been established, although several types of therapies for MRONJ have been tried. However, dentists have to perform daily dental treatment in patients who receive high-risk medications for developing MRONJ. Therefore, available clinical information about what we should do when performing dental treatments in osteoporosis and cancer patients receiving antiresorptive agents or other medications is imperative to prevent development of MRONJ. Thus, the aims of this study were to (1) comprehensively review the current definitions, epidemiology, risk factors, and staging of and treatment strategies for MRONJ; and (2) provide clinical considerations or recommendations and precautions when dental treatment is performed in patients receiving high-risk medications for developing MRONJ to prevent its occurrence. The null hypothesis for this study was that “clinical considerations or recommendations for dental treatment are similar between in patients prior to or receiving high-risk medications for developing MRONJ and healthy patients not using these medications,” even though this was a literature review without any statistical analysis and was not a systematic review and meta-analysis.

## Materials and methods

### PICO question

The question in this literature review was formatted according to the Population, Intervention, Comparison, and Outcomes (PICO) framework, as follows:

• Population: Human subjects

• Intervention: Dental treatment in patients prior to or receiving high-risk medications for developing MRONJ

• Comparison: Dental treatment in healthy patients not taking high-risk medications for developing MRONJ

• Outcome: Clinical considerations or recommendations for dental treatment

### Literature search strategy

A literature search was conducted on PubMed. The following search terms were used: “osteonecrosis,” “osteonecrosis of the jaw,” “jaw necrosis,” “bisphosphonate(s),” “denosumab,” “RANKL,” “medication,” “antiresorptive(s),” “antiresorptive agent,” “literature review,” “review,” “systematic review,” “statement,” and “guideline.” The PubMed search was independently performed by the two reviewers (M.K. and S.K.). The inclusion criteria were as follows: (1) articles published in English or German from December 2014 to December 2020; (2) human studies; and (3) review articles, systematic reviews, systematic reviews and meta-analyses, consensus statements, and guidelines. The exclusion criteria were as follows: (1) animal studies; and (2) in vitro studies. The related entry key words were used in different combinations using the Boolean operators “AND” and “OR” for the PubMed search as follows: #1 (“osteonecrosis” OR “osteonecrosis of the jaw” OR “jaw necrosis”) AND #2 (“bisphosphonate(s)” OR “denosumab” OR “RANKL” OR “medication” OR “antiresorptive” OR “antiresorptive agent”) AND #3 (“literature review” OR “review” OR “systematic review” OR “systematic review and meta-analysis” OR “statement” OR and “guideline”). Moreover, a manual search was also conducted in regard to all references from selected articles and other articles identified on the PubMed search, irrespective of publication date, and inclusion and exclusion criteria.

Studies were investigated by screening the titles and abstracts, and included in this study by final discussion between SK and MK. Additional reviewer (TS) was also consulted for agreement when 2 independent reviewers disagree the results of screening. A total of 161 articles that fulfilled the inclusion criteria on the PubMed search were selected. Seventy-five of the articles were systematic reviews with or without a meta-analysis, 2 articles were consensus statements, 5 articles were clinical guidelines, and 33 were other types of research articles. After title and abstract screening, a total of 22 articles were selected for full-text review in addition to 53 articles selected by manual search.

## Results

### Current definition and staging of MRONJ

First of all, a correct diagnosis of MRONJ is needed. Lesions in the maxillofacial region that fulfill the following clinical situations are diagnosed as MRONJ: “(1) exposed bone in the maxillofacial region that does not heal within 8 weeks after identification by a health care provider; (2) current or previous treatment with antiresorptive or antiangiogenic agents; and (3) no history of radiation therapy to the jaws or obvious metastatic disease to the jaws” [1, 2, 9].

A well-established optimal staging system should be used after the diagnosis of MRONJ, since appropriate staging of MRONJ based on clinical symptoms and radiographic findings helps clinicians provide appropriate treatment strategies for MRONJ patients [1, 2, 10] (Tables 1 and 2), although there is no clinical evidence regarding which staging systems are recommended due to no validation of the different systems [10]. The setting of stage 0 has been controversial, since it has been reported that 50% of them developed MRONJ in clinical situations, although the remaining of 50% may not progress to clinically evident MRONJ [10, 11].

**Table 1:** Clinical and imaging findings of MRONJ in each stage

Staging	Clinical symptoms	Imaging findings
*Stage 0*	no bone exposure/necrosis, deep periodontal pocket, loose tooth, oral mucosal ulcer, swelling, abscess formation, trismus, hypoesthesia/numbness of the lower lip (Vincent’s symptom), non-odontogenic pain	sclerotic alveolar bone, remaining tooth extraction socket, alveolar bone loss or resorption not attributable to chronic periodontal disease, changes to trabecular pattern—dense bone and no new bone in extraction sockets, regions of osteosclerosis involving the alveolar bone or surrounding basilar bone, thickening or obscuring of the periodontal ligament (thickening of the lamina dura, sclerosis, and decreased periodontal ligament space)
*Stage 1*	asymptomatic bone exposure/necrosis without signs of infection, or fistula in which the bone is palpable with a probe	
*Stage 2*	bone exposure/necrosis associated with pain, infection, fistula in which bone is palpable with a probe or at least one of the following symptoms including bone exposure/necrosis over the alveolar bone, which result in pathologic fracture, extraoral fistula, nasal/maxillary sinus fistula formation, or advanced osteolysis extending to the mandibular inferior edge or maxillary sinus	
*Stage 3*	bone exposure/necrosis associated with pain, infection, or at least one of the following symptoms, or fistula in which bone is palpable with a probe. Bone exposure/necrosis over the alveolar bone. As a result, pathologic fracture or extraoral fistula, nasal/maxillary sinus fistula formation, or advanced osteolysis extending to the mandibular inferior edge or maxillary sinus	osteosclerosis/osteolysis of the surrounding bone (cheek bone, palatine bone), pathologic mandibular fracture, and osteolysis extending to the maxillary sinus floor

**Table 2:** Staging and treatment strategies for MRONJ

Staging	Clinical symptoms
*Stage 0*	systemic management, including use of pain medications and antibiotics improvement of oral hygiene (rinsing and cleaning of fistulas and periodontal pockets)
*Stage 1*	antibacterial mouth rinse clinical follow-up on a quarterly basis improvement of oral hygiene (rinsing and cleaning of fistulas and periodontal pockets) patient education and review of indications for continued bisphosphonate therapy
*Stage 2*	combination or monotherapy of symptomatic treatment with oral antibiotics and/or oral antibacterial mouth rinse pain control debridement to relieve soft tissue irritation and infection control
*Stage 3*	antibacterial mouth rinse antibiotic therapy and pain control surgical debridement or resection for longer-term palliation of infection and pain extraction of tooth in exposed bone/necrotic bone as source of infection maintenance of nutrition with supplements and infusions

### Epidemiology of MRONJ

Recently, evidence regarding the epidemiology of MRONJ has been accumulated. In this section, the prevalence and incidence rates of BRONJ, DRONJ, and other MRONJs are described by the type of drug administered (Table 3).

**Table 3:** Epidemiology of MRONJ

MRONJ-inducing drugs	Prevalence	Incidence
*Oral bisphosphonates Intravenous bisphosphonates*	<0.001% (0% to 0.04%) 1.0% to 2.3% for 3-year administration	1.04 to 69 per 100,000 patient-years 0 to 12,222 per 100,000 patient-years
*Anti-RANKL antibody Osteoporosis Cancer*	1.3% to 3.2% for 3-year administration	0 to 30.2 per 100,000 patient-years 0 to 2,316 per 100,000 patient-years
*Angiogenic inhibitors*	0.3% to 0.4%	
*Anti-sclerostin antibody*	Unknown	Unknown
Anti-TNFα antibody		
Anti-CD20 antibody		
Other monoclonal antibodies		
Tyrosine kinase inhibitors		
mTOR inhibitors		

#### High-dose bisphosphonate and denosumab users with cancers

The latest systematic review and meta-analysis with 13,857 patients reported that the prevalence of BRONJ in cancer patients receiving zoledronic acid (Zol) ranged from 0.4% to 1.6%, 0.8% to 2.1%, and 1.0% to 2.3% after 1, 2, and 3 years of Zol exposure, respectively, whereas the prevalence of DRONJ in cancer patients receiving denosumab ranged from 0.5% to 2.1%, 1.1% to 3.0%, and 1.3% to 3.2% after 1, 2, and 3 years of denosumab exposure, respectively [12]. The study also showed that DRONJ occurred significantly more often than BRONJ in cancer patients after 1, 2, and 3 years of exposure to antiresorptive agents, although the prognosis of DRONJ was similar to that of BRONJ [12]. The International Task Force on Osteonecrosis of the Jaw has reported that the incidence of BRONJ in cancer patients treated with intravenous (IV) bisphosphonates including Zol ranged from 0 to 12,222 per 100,000 patient-years, whereas the incidence of DRONJ in cancer patients treated with denosumab ranged from 0 to 2,316 per 100,000 patient-years [2].

#### Low-dose bisphosphonate and denosumab users with osteoporosis

It has been estimated that the prevalence of antiresorptive agent-related ONJ (ARONJ) in osteoporosis patients ranges from 0.001 to 0.01% in Australia, Canada, Germany, the USA, and Sweden. It has been reported that the prevalence of BRONJ in osteoporosis patients taking oral bisphosphonates ranges 0% to 0.04%, although it is usually less than 0.001% [2, 9, 13–15]. However, there have been limited data on the prevalence of DRONJ.

The incidence of BRONJ in patients taking oral bisphosphonates for the treatment of osteoporosis ranges from 1.04 to 69 per 100,000 patient-years, whereas in osteoporosis patients receiving IV bisphosphonates, it ranges from 0 to 90 per 100,000 patient-years. On the other hand, the incidence of DRONJ in patients receiving denosumab for treatment of osteoporosis ranges from 0 to 30.2 per 100,000 patient-years. Therefore, the incidence of ARONJ in osteoporosis patients is relatively very low, regardless of the types of antiresorptive agents [2].

#### Angiogenic inhibitor users

Guarneri et al have reported that the prevalence of ONJ in advanced breast cancer patients receiving an angiogenic inhibitor, monoclonal antibody to VEGF (bevacizumab), ranged from 0.3 to 0.4%, whereas the prevalence with bevacizumab and bisphosphonate combination therapy ranged from 0.9 to 2.4% [16].

#### Other medication users

One systematic review and one review have reported on MRONJ unrelated to antiresorptive agents and angiogenic inhibitors [7, 17], although there were not descriptions regarding the incidence and prevalence of MRONJ unrelated to them. In 2019, 30 tyrosine kinase inhibitors were approved. Between 2010 and 2014, 418 cases of ONJ induced by tyrosine kinase inhibitors (sunitinib, sorafenib, pazopanib, and axitinib) were reported to the U.S. Food and Drug Administration [18]. Other tyrosine kinase inhibitors such as imatinib [19], regorafenib [20], and cabozantanib [21] have also been reported to possibly cause MRONJ. Monoclonal antibodies (rituximab, adalimumab, infliximab, and romosozumab) have also been reported to possibly induce MRONJ, as well as denosumab and bevacizumab. Two cases by rituximab [22, 23], 4 cases by adalimumab and infliximab, which are monoclonal TNF-α antibodies [24], and 2 cases by romosozumab, which is a human monoclonal sclerostin antibody [25], have been reported to possibly cause MRONJ. mTOR inhibitors (everolimus and temsirolimus) and immunosuppressants (methotrexate and corticosteroids) may also cause MRONJ. However, caution is needed when interpreting MRONJ cases unrelated antiresorptive drugs and angiogenic inhibitors, since MRONJ cases caused by the above-mentioned medications are extremely rare [7, 17].

### Triggering factors and risk factors for MRONJ, comorbidities, and medications

#### Triggering factors for MRONJ

A recent systematic review investigating 3198 cases of BRONJ reported the triggering factors for BRONJ, regardless of administration routes, with 61.7%, 14.8%, 7.4%, 7.2%, 5.0%, and 3.9% of BRONJ triggered by tooth extraction, spontaneous onset, prosthesis-induced trauma such as ill-fitting dentures, history of dental surgery, periodontitis, and dental implant-related treatment, respectively [26].

On the contrary, there is limited information about the triggering factors for DRONJ. Two studies have reported that tooth extraction, poor oral hygiene, or dental appliances such as removable prostheses are among potential triggering factors for DRONJ [27, 28]. Currently, no clinical information on the triggering factors for MRONJ unrelated to antiresorptive agents is available.

#### Risk factors for MRONJ

Based on the recent review and systematic review of the risk factors for MRONJ, statements of the International Task Force on ONJ, the Position paper on ONJ of the American Association of Oral and Maxillofacial Surgeons (AAOMS), and the Position paper 2017 of the Japanese Allied Committee on ONJ [1, 2, 9, 29, 30], the following local risk factors have been identified. As mentioned above, IV and oral bisphosphonates and denosumab are undoubtedly risk factors for developing MRONJ [1, 2, 9, 29, 30]. Moreover, angiogenesis inhibitors, tyrosine kinase inhibitors, monoclonal antibodies, mTOR inhibitors, and immunosuppressants have been reported as possible risk factors for the development of MRONJ [7, 16, 17, 24] (Table 4).

**Table 4:** Risk factors for MRONJ

Risk factors		
Local risk factors	Anatomical factors	mandible rather than maxilla maxillary or mandibular tori exostoses knife-edge ridgemylohyoid ridge
	Dental treatment	tooth extraction implant treatment (placement, bone augmentation, periimplantitis, removal) periodontal surgery endodontic treatment (especially apicectomy) other oral surgery except for above-mentioned risk factors
	Dental prosthesis	fixed prostheses (non-passive fit) ill-fitting dentures
	Other oral conditions	excessive bite force poor oral hygiene xerostomia
Systemic risk factors	Medications	chemotherapy for malignant tumors corticosteroids
	Systemic diseases	oncology patients receiving IV bisphosphonates or high-dose denosumab diabetes osteoporosis rheumatoid arthritis cardiovascular disease (hypertension, hyperlipidemia and angina) Sjögren’s syndrome sarcoidosis hypocalcemia hypoparathyroidism osteomalacia vitamin D deficiency renal dialysis anemia Paget’s disease of bone
	Others	tobacco use alcohol intake obesity advanced age
Other risk factors	Genetic factors	single-nucleotide polymorphisms (CYP2C8, SIRT1/HERC4)

#### Local risk factors

Tooth extraction is the most often reported risk factor for MRONJ. MRONJ occurs more commonly in the mandible than in the maxilla (65% vs. 28.4% in the mandible vs. maxilla, 6.5% in both), although reasonable biological mechanisms remain unknown. Moreover, periodontal disease, acute dental infection, dental implant treatment (implant placement, bone augmentation, peri-implantitis, and removal), periodontal surgery, other oral surgeries, endodontic treatment, removable and fixed dental prostheses, trauma induced by ill-fitting dentures, anatomical factors (maxillary and mandibular tori, exostoses, knife-edge ridge, and mylohyoid ridge), and other oral conditions (excessive bite force, poor oral hygiene, and xerostomia) have also been implicated [1, 2, 9, 29–31].

#### Systemic risk factors

Chemotherapy for malignant tumors (multiple myeloma, and breast, prostate, lung, renal and colon cancers), corticosteroid use, diabetes, tobacco use, and cardiovascular disease (hypertension, hyperlipidemia, and angina) are the most commonly reported systemic risk factors. Oncology patients receiving IV bisphosphonates or high-dose denosumab are at risk for the development of MRONJ. Osteoporosis, rheumatoid arthritis, Sjögren’s syndrome, sarcoidosis, hypocalcemia, hypoparathyroidism, osteomalacia, vitamin D deficiency, renal dialysis, anemia, Paget’s disease of bone, erythropoietin therapy, cyclophosphamide therapy, alcohol intake, and obesity have also been reported to be systemic risk factors for developing MRONJ [1, 2, 9, 29, 30].

In a study of 2674 patients diagnosed with BRONJ, they had the following comorbidities and medications: chemotherapy (39.7%), corticosteroid therapy (24.6%), diabetes mellitus (11.2%): hypertension (8.0%), smoking habit (8.0%), thrombin coagulopathies (4.0%), and no concomitant diseases (4.1%) [26]. On the other hand, denosumab-treated patients diagnosed with DRONJ have been reported to receive chemotherapy (64% [27] and 75% [28] of patients with DRONJ) or angiogenic inhibitors (20% of patients with DRONJ) [28].

It has also been well documented that advanced age is one of the significant risk factors for developing BRONJ in bisphosphonate users, regardless of administration route [2, 32, 33]. A recent report showed that MRONJ occurred most commonly in older patients (median age 60 years) [17]. Therefore, caution should be exercised when dental treatment is performed in patients more than 60 years old.

#### Other risk factors

It has been reported that genetic factors such as single-nucleotide polymorphisms (SNPs; CYP2C8 and SIRT1/HERC4) may become risk factors for MRONJ, although a recent systematic review concluded that SNPs were not correlated with the developing MRONJ [34]. Root canal treatment and orthodontic treatment are not risk factors for MRONJ [30].

### Clinical considerations in dental treatment for the prevention of MRONJ

#### Cancer patients prior to initiation of high-dose antiresorptive agents [1, 9, 10, 30, 35]

It has been well documented that cancer patients receiving powerful antiresorptive therapy are among those at highest risk for the development of BRONJ/DRONJ. Therefore, if the patient’s systemic condition permits, prior to initiation of powerful antiresorptive therapy, a good oral condition should be provided by comprehensive dental assessment including radiographic dental, periodontal, and radiographic examinations. Patient education for the maintenance of good oral hygiene and regarding the risk factors for the development of ARONJ is also important. Appropriate management of patients prior to high-dose antiresorptive therapy decreases the incidence of BRONJ/DRONJ [35–38].

Unrestorable and/or poor prognosis teeth should be extracted. Other necessary elective dentoalveolar surgery should also be completed. Antiresorptive therapy may be started after epithelial wound coverage is completed if the systemic condition permits. In humans, epithelial wound coverage is complete in 14 to 21 days. In addition to denture-induced ulcers on mucosa, especially the areas around the mylohyoid line and the maxillary or mandibular torus, the fitting of complete dentures and removable partial dentures should be examined when patients use either or both of them. Their relining or new fabrication should be performed prior to initiation of antiresorptive therapy when the dentures are clinically judged as ill-fitting. Ideally, placement of dental implants should be completed before the administration of high-dose antiresorptive agents, although nonurgent surgical procedures should be delayed if necessary [2, 9]. Cessation of smoking is strongly recommended. Uncontrolled systemic diseases that are risk factors for developing BRONJ/DRONJ should be under control, if possible, prior to initiation of antiresorptive therapy. The start date of antiresorptive therapy must be decided by the oncologist or physician after dentists communicate fully with them regarding dental treatment. Both physicians and dentists must explain the benefits of antiresorptive therapies and the risks of BRONJ/DRONJ to patients before anti-resorptive therapy. However, high-dose antiresorptive therapy may be acceptable in parallel with dental education, examination, and treatment when high-dose antiresorptive therapy cannot be delayed due to progression of bone metastasis and the need for therapy to inhibit SREs [30].

#### Osteoporosis patients prior to initiation of antiresorptive agents [1, 9, 10, 30, 35]

Osteoporosis is well known as one of the risk factors for developing BRONJ/DRONJ, although the incidence of BRONJ/DRONJ is relatively low in oral bisphosphonate or denosumab users for osteoporosis treatment compared to the incidence in high-dose IV bisphosphonate or denosumab users with malignant tumors. Clinical considerations for MRONJ in osteoporosis patients taking oral bisphosphonates and subcutaneous denosumab are basically the same for those in cancer patients receiving powerful antiresorptive agents. In particular, dentists should educate patients that the risk of developing BRONJ seems to increase when administration duration exceeds 3 or 4 years in oral bisphosphonate users [1, 39], since long-term treatment periods are generally required for osteoporosis. The same point should be made to denosumab users for osteoporosis, although there is limited information on the relationship between administration duration of denosumab and the risk of developing DRONJ.

#### Cancer and osteoporosis patients receiving antiresorptive therapy

To suppress and decrease the potential risk of the development of ARONJ, some clinical recommendations to prevent ARONJ have been reported in position papers, guideline, consensus statements, or reviews [1, 2, 10, 30, 35, 40]. In this section, some clinical protocols for the prevention of ARONJ are introduced, although all of them do not always have high-level scientific evidence. First of all, maintaining good oral hygiene and dental care is the most important measure to prevent new pathological lesions possibly triggering ARONJ. Dental education should also be continued.

##### Surgical treatment including tooth extraction in antiresorptive agent users

Tooth extraction has been well documented to be one of the most serious risk factors for developing ARONJ [1, 2, 10, 30, 35]. The estimated risk for developing BRONJ after tooth extraction in cancer patients receiving IV bisphosphonates ranges from 1.6 to 14.8% [1, 10]. Most recently, it has been recommended that dentists start invasive dental treatment without discontinuation of IV or oral bisphosphonates [10, 30], although it has been previously recommended that non-restorable teeth may be treated by removal of the crown and endodontic treatment of the remaining roots without extraction in cancer patients receiving IV bisphosphonates [1]. Perioperative administration of antibacterial agents is recommended when tooth extraction is performed. Moreover, it has been recommended that smoothing the remaining sharp edges of alveolar bone and wound closure with mucoperiosteal flaps lined by periosteum should be performed [30]. On the other hand, there is limited information about the relationship between tooth extraction and denosumab users. However, a recent clinical guideline and the Japanese position paper have recommended that invasive dental treatment including tooth extraction could be performed without a drug holiday [10, 30].

##### Dental implant treatment in antiresorptive agent users

Placement of dental implants, removal of dental implants, peri-implantitis, and taking oral bisphosphonates after implant placement have been reported to trigger the induction of ARONJ in osteoporosis or cancer patients [30, 31, 41–44]. The differences and the degrees of risk for developing ARONJ among these implant-related events have not been fully elucidated, since scientific and clinical data are limited. However, based on our current knowledge, administration route (oral vs. intravenous), dose (low-dose vs. high-dose), duration (short-term vs. long-term), and surgical site (maxillae vs. mandible, or anterior vs. posterior sites) may be associated with the development of ARONJ triggered by implant-related events. Implant removal should not be considered in cancer or osteoporosis patients receiving oral and/or intravenous bisphosphonates with peri-implantitis, since implant removal may possibly be one of the risk factors for developing ARONJ [31]. Strict maintenance programs such as short-term recall intervals, oral examinations of soft and hard tissues around natural teeth and dental implants, radiographical evaluations, and professional cleaning of peri-implant tissue are highly recommended.

The AAOMS has proposed that the risk for developing ARONJ in implant treatment is considered to be comparable to that in tooth extraction [1]. Implant-related surgical procedures are contraindicated in cancer patients receiving high-dose antiresorptive agents. On the other hand, implant treatment may be allowed without a drug holiday in osteoporosis patients receiving oral bisphosphonates or denosumab [1, 9, 30, 35]. In particular, the AAOMS has recommended that clinical information about possible implant failure with the risk for developing BRONJ should be provided when implant treatment is performed in osteoporosis patients receiving oral bisphosphonates for less than 4 years with no other medical risk factors, although the risks of implant failure and development of ARONJ are relatively low [1, 35]. On the contrary, there are no scientific data for clinical recommendations about implant treatment in osteoporosis patients taking oral bisphosphonates for less than 4 years with other medical risk factors and for more than 4 years with or without any medical risk factors [1]. There is a lack of information about whether implant treatment could be performed. Therefore, caution should be exercised when dental implant treatment is planned for osteoporosis patients taking oral bisphosphonates for more than 4 years, for less than 4 years with medical risk factors, or for osteoporosis patients receiving denosumab, regardless of administration period.

##### Periodontal therapy in antiresorptive agent users

Periodontal disease has been well documented as a risk factor for the development of ARONJ [1, 2, 9, 10, 26, 30]. Recently, a systematic review and meta-analysis has also shown that periodontitis poses a higher risk for developing MRONJ, with a risk ratio of 2.75 (95% confidence interval: 1.67–4.52) [45]. Thus, periodontal treatment is required for patients receiving antiresorptive agents to prevent and decrease the risk of ARONJ. However, periodontal surgery has been reported to be considered a risk factor for developing ARONJ, similar to tooth extraction [1]. Non-surgical treatment including pocket probing and scaling and root planning for periodontal disease is recommended.

##### Prosthetic treatment in antiresorptive agent users

There has been clinical evidence that wearing of ill-fitting dentures is one of the risk factors for the development of ARONJ [1, 2, 9, 10, 30, 35]. Therefore, removable partial and full dentures should be examined and evaluated when patients receiving antiresorptive agents visit dental clinics, even when patients have no chief complaint related to wearing dentures [1]. New fabrication or relining of dentures should be performed for denture stability [1, 35]. Fixed partial dentures or single crowns with precise margins may be fabricated, although whether they are the risk factors for developing ARONJ remains unclear [46].

##### Endodontic treatment in antiresorptive agent users

Endodontic treatment is not considered to be a risk factor for developing ARONJ [30]. However, apicoectomy has been considered a risk factor for developing ARONJ with the same level of risk as tooth extraction [1]. Moreover, periapical lesions have also been considered to be risk factors for the development of ARONJ [1, 47]. Therefore, to resolve periapical lesions, root canal treatment may proceed to prevent the development of ARONJ in antiresorptive users, although apicectomy should be avoided [35].

##### Orthodontic treatment in antiresorptive agent users

There has been no evidence to establish that orthodontic treatment is a risk factor for developing ARONJ. Therefore, in current clinical situations, orthodontic treatment is not considered to be a risk factor for developing ARONJ [30]. Dentists can start orthodontic treatment in antiresorptive agent users, although it is difficult to move teeth ideally due to the effects of antiresorptive drugs. However, caution should be taken when patients receiving antiresorptive drugs, for whom orthodontic treatment is planned to start, have other risk factors for the development of ARONJ.

#### Patients prior to initiation of or receiving other medications for developing MRONJ

There have been no evidence-based clinical recommendations for the prevention of MRONJ induced by medications other than antiresorptive drugs. Basically, clinical recommendations for prevention of MRONJ may follow those in antiresorptive users proposed by the position papers, guidelines, consensus statements, or reviews [1, 2, 10, 30, 35, 40]. More clinical evidences is needed to establish preventive protocols for MRONJ.

### Treatment strategies for MRONJ

There is limited evidence regarding the mechanisms of MRONJ, although several animal MRONJ models have been developed to address them [48–58]. Therefore, current treatment strategies for MRONJ have been constructed based on clinical aspects rather than scientific data. In this section, the treatment strategy for MRONJ at each stage is introduced based on several position papers [1, 14, 30, 35], a clinical guideline [10], and other studies including systematic reviews and consensus statements by the International Task Force on ONJ [2, 9, 26, 41, 59].

#### At risk

No treatment is needed for the “*At risk*” stage, which is characterized by patients who have or had been treated with IV or oral antiresorptive or antiangiogenic therapy. However, patient education regarding MRONJ (prevalence, risks, and symptoms), maintenance of good oral hygiene, and reduction of modifiable risk factors is required to avoid development of MRONJ [1, 10, 26, 35, 59].

#### Stage 0

It has been reported that 50% of patients in “*Stage 0*” have progressed to stage 1, 2, or 3 [11]. Some reports have discussed *Stage 0* with BRONJ patients [11, 60, 61]. The AAOMS [1], the Multinational Association of Supportive Care in Cancer/International Society of Oral Oncology (MASCC/ISOO) and the American Society of Clinical Oncology (ACSO) [10], the Japanese Allied Committee on ONJ [30], and the Korean Society for Bone and Mineral Research and the Korean Association of Oral and Maxillofacial Surgeons have included *Stage 0* in staging [35], although the International Task Force on ONJ has not included it in the staging of MRONJ due to the potential risk of overdiagnosis [2]. Therefore, caution should be taken when diagnosing patients who have some symptoms without exposed bone as *Stage 0* MRONJ, due to overdiagnosis. However, symptomatic treatment (chronic pain and infection control by appropriate medications) and conservative management are recommended for patients with *Stage 0*. A close follow-up system should be created for the early detection of progression to a higher stage of MRONJ [1, 9, 10, 26, 30, 59].

#### Stage 1

Conservative therapy (e.g., the use of antimicrobial oral rinses, monitoring patients with *Stage 1* four times a year, improvement of oral hygiene) is recommended along with continuing patient education. Communication between the oncologists and/or physicians and dentists is strongly encouraged. Immediate surgery is not required at this stage, although minor surgical procedures such as sequestration or removal of necrotic bone are recommended to reduce soft tissue trauma [1, 9, 10, 26, 30, 35].

#### Stage 2

Treatment strategies for MRONJ *Stages 2* and *3* are difficult to distinguish clearly, since some position papers, clinical reviews, and a clinical guideline recommend conservative rather than surgical treatment. In the following sections, information about treatment strategies for MRONJ *Stages 2* and *3* are provided based on position papers, a clinical guideline, and systematic reviews, without any personal opinions.

In addition to antimicrobial mouth rinses, systemic antimicrobial control is recommended with antibiotics, although there is limited clinical evidence regarding which antibiotics are better for patients with *Stage 2* MRONJ [1, 9, 10, 26, 30, 35]. Pain control and removal or debridement of necrotic bone may be considered, since formation of a bacterial membrane on exposed bone has been reported to inhibit the efficacy of systemic antimicrobial therapy [62–64]. Follow-up every 8 weeks and patient education are also recommended based on the guideline for MRONJ [10].

#### Stage 3

As with other *stages* of MRONJ, treatment revolves around antimicrobial mouth rinse and systemic antibiotic therapy with pain control. However, the necessity for extensive treatment is greater in many MRONJ cases. Surgical removal of a superficial and well-defined sequestrum should be considered when adjacent soft tissue is irritated by the sequestrum. Moreover, resection of a sequestrum, partial resection of maxillary bone, or hemi-mandibular resection should be performed for longer-term symptom amelioration. Referral to surgeons and/or oncologists may be needed when performing surgical procedures, since the bone lesions that are planned for removal, debridement, or resection may include cancer metastasis [1, 9, 10, 26, 30, 35].

#### Surgical treatment for MRONJ

As already described, some position papers, clinical reviews, and a clinical guideline have recommended non-surgical treatment strategies for amelioration and/or resolution of MRONJ [1, 9, 10, 26, 30, 35]. However, recently, a systematic review demonstrated that conservative and extensive surgical approaches improved the healing rate of MRONJ when compared with a nonsurgical approach [41]. The study reported that the healing rates of S*tages 1*, *2*, and *3* with a nonsurgical approach were 33%, 24%, and 0%, respectively. On the other hand, the authors have demonstrated that the healing rates of MRONJ with conservative surgical and extensive surgical approaches were 72% and 87% for *Stage 1*, 79% and 96% for *Stage 2*, and 27% and 81% for *Stage 3*, respectively. Therefore, a surgical approach, rather than a nonsurgical approach, may be considered first if the patient’s systemic condition permits. However, the complete healing rate of MRONJ in cancer patients receiving high-dose antiresorptive therapy has been reported to be approximately 50%, even though a surgical approach was chosen as a treatment strategy for MRONJ [65], which suggests that caution should be exercised, since a surgical approach is not always superior to a nonsurgical approach for treatment for MRONJ lesions.

### Cessation of antiresorptive drugs

First of all, dentists should not decide the cessation of high-dose antiresorptive agents, known as a drug holiday, in the patients who have serious clinical conditions such as cancer or immunosuppression, since discontinuation of antiresorptive agents has a potential risk of the relapse of SREs or tumor induced hypercalcemia. There is limited evidence that cessation of antiresorptive agents contributes to preventing BRONJ/DRONJ in many position papers and a guideline [1, 9, 10, 30, 35, 38], although they have reported that cessation of antiresorptive agents is controversial.

The half-life of bisphosphonates has been reported to be more than 10 years due to the higher affinity to hydroxyapatite, although that of denosumab is about 26 days after administration [66]. Moreover, the European Calcified Tissue Society has concluded that denosumab should not be discontinued without consideration of alternative treatment [67].

On the other hand, many reports have assessed the effects of discontinuation of antiresorptive agents on the resolution and/or amelioration of BRONJ/DRONJ. Most studies have demonstrated no effects on the clinical outcomes by cessation of antiresorptive agents [68–73], whereas two of them reported that a drug holiday had a positive effect on healing of BRONJ/DRONJ [74, 75].

Therefore, theoretically, given the above-mentioned reasons, taking into account the risks and benefits of discontinuation of antiresorptive agents with respect to systemic conditions, cessation of antiresorptive agents for the prevention or healing/amelioration of BRONJ/DRONJ is basically not recommended.

## Conclusions

Within the limitation of this literature review due to no systematic review and meta-analysis, the current status of MRONJ, such as epidemiology, risk factors, and staging of and treatment strategies, was described based on the clinical guidelines for MRONJ, the position papers on MRONJ, consensus statements, and literature reviews. Some clinical recommendations for each dental treatment in osteoporosis patients and cancer patients prior to or currently receiving antiresorptive agents were also described. There is a certain level of clinical evidence supporting the clinical recommendations for bisphosphonate users, whereas there is limited evidence for denosumab, angiogenesis inhibitors, and other medications. The mode of action of each drug is quite different, although the clinical characteristics of MRONJ are similar among drugs. Therefore, caution should be exercised when dental treatment is performed for users of denosumab, angiogenesis inhibitors, and other medications. Further clinical evidence needs to be accumulated to adequately create clinical recommendations/guidelines for patients at higher risk of MRONJ.

## Data Availability

The datasets used and/or analyzed during the current study are available from the corresponding author on reasonable request.

## Abbreviations

MRONJ: Medication-related osteonecrosis of the jaw
BRONJ: Bisphosphonate-related osteonecrosis of the jaw
DRONJ: Denosumab-related osteonecrosis of the jaw
ARONJ: Antiresorptive agent-related osteonecrosis of the jaw
SREs: Skeletal-related events
Zol: Zoledronic acid
VEGF: Vascular endothelial cell growth factor
TNF: Tumor necrosis factor
AAOMS: The American Association of Oral and Maxillofacial Surgeons
